# The current consensus on ulcerative colitis, and the evidence and perspectives on the influence of gut microbiota on it

**DOI:** 10.3389/fmed.2026.1735850

**Published:** 2026-01-27

**Authors:** Yufeng Chen, Chang Liu

**Affiliations:** 1 The First People’s Hospital of Jiashan County, Jiaxing, Zhejiang, China; 2 Reproductive Medicine Center, The First Affiliated Hospital of Wenzhou Medical University, Wenzhou, Zhejiang, China

**Keywords:** gut microbiota, inflammatory bowel disease, pathogenesis, therapeutic strategies, ulcerative colitis

## Abstract

Ulcerative colitis (UC) is a chronic non-specific inflammatory disease, the pathogenesis is not clear, there is no clinical cure. The number of cases of UC has increased worldwide in recent years due to industrialization and social pressures. At present, the therapeutic effectiveness of UC remains controversial. Although researchers have conducted certain studies on the pathogenesis of UC, its pathogenesis and anti-UC pathogenesis have not been fully revealed. Previous studies have found a close relationship between human gut microbes and UC, and may be the most important measure of UC for clinical judgment. Many studies have linked UC to disruption of the gut microbiome, which is one of the most important features of UC. This paper reviews the clinical characteristics, pathogenesis and current treatment strategies of UC, and reviews the interaction between intestinal flora and UC as well as the therapeutic effects of intestinal flora, providing reference for the prevention and treatment of UC.

## 1 Introduction

One of the two types of inflammatory bowel diseases (IBD) is UC, and the other is Crohn’s disease (CD), which affects millions of individuals worldwide (1). Among them, UC has a high incidence and low cure rate, new drugs have been used to heal the colonic mucosa. The mucosal surface is the primary site of UC, throughout the colon, beginning in the rectum and extending proximally and continuously. There are several clinical manifestations of UC, including mucous, pus, blood stools, abdominal pain, diarrhea, and liesia. There is a long course of UC, the disease is recurrent, and it is difficult to control, which seriously affects patients’ physical and mental health.

The pathogenesis of UC has not been definitively determined, but it is certain that it is affected by environmental, individual differences, genetics, immune disorders, gut microbiota disorders and many other factors. Industrialization and social pressure have contributed to an increase in UC incidence in recent years. Western medicine for the treatment of this disease is mainly immunosuppressive, anti-inflammatory drugs, the treatment effect of acute stage patients is obvious, but long-term repeated use of Western medicine will cause more adverse reactions, resulting in patients with reduced sensitivity to drugs, the disease is easy to repeat after drug withdrawal. However, treatment from the perspective of gut microbiota seems to have the advantages of less adverse reactions and significant curative effect. From the perspective of intestinal microecology, this paper examines UC in recent years, gut microbiota composition and function, as well as their relationship, were discussed.

## 2 Ulcerative colitis

### 2.1 Definition and clinical presentation of UC

A continual nonspecific IBD, UC has no recognized cause. It is related with CD, additionally acknowledged as IBD (2). UC affords as localized infection on the mucosal floor of the colon. In most cases, the disorder starts off evolved in the rectum and extends proximally via the colon, but infection of the cecum may additionally take place in sufferers with proctitis and left colitis. Proctitis, left colitis, and pervasive colitis are stratified according to the degree of colon involvement (3).

Diarrhea, mucous and bloody stools, abdominal pain, and mucous and bloody stools are the most common clinical manifestations of UC. In more severe cases, symptoms such as fever and weight loss may accompany them. The most common symptom is diarrhea. According to the statistics of Peking Union Medical College Hospital in China from 1974 to 2007, about 95.7% of UC patients are accompanied by diarrhea. However, acute UC can be manifested as alternating constipation and diarrhea (4, 5). And blood in the stool is second only to diarrhea symptoms, due to a high proportion of rectal involvement, often manifested as anal fall and distension, stool meaning frequent but poor defecation (6). There are also some patients with abdominal pain, mostly located in the left lower abdomen dull pain, or intermittent abdominal cramps, with the characteristics of relief after the stool, more serious patients with abdominal pain symptoms, and the increase of the lesion intestinal wall tension related. In addition, UC patients are accompanied by many extra-gastrointestinal symptoms, such as joint complications, peripheral arthropathy and axial arthropathy (7). The skin showed erythema nodosum, pyoderma gangrenosum and nonspecific rash. Ocular manifestations were scleritis and uveitis. Nonspecific fatty liver, cholelithiasis and primary sclerosing cholangitis (5).

Clinical observation of a large number of UC patients, it was found that the initial lesion of the intestinal tissue of patients with UC was manifested as the infiltration of neutral multinucleated cells and round cells in the basement crypt of the mucosa, resulting in crypt abscess (8). The swelling of mitochondria, the dilatation of intercellular space and the dilatation of inner plasma can be seen clearly in electron microscope (9). As the disease progresses, the crypt abscess and covering epithelium become detached, leading to ulcers. The ulcer is adjacent to a relatively normal mucosa, but the mucosa is edema, which gives it a polypoid appearance and is isolated between adjacent ulcers. The ulceration area is occupied by the indulgent growth of granulation and collagen tissue, and the ulceration is aggravated, but penetration of the musculature is less common. In toxic megacolon and explosive UC such lesions may penetrate the entire intestinal tissue and present as intestinal perforation, but these lesions are rare. The patient has about 20 bloody stoes every day. Due to the light peeling of the intestinal wall and the obviously deformed mucous membrane cannot effectively absorb water and sodium, every peristalsis will squeeze out a large amount of blood from the exposed granulation tissue surface, so the mucosal abscess and blood of this disease is more obvious than that of Rohn’s disease (10–12).

### 2.2 Pathogenesis of UC

UC, as a recurrent and refractory digestive disease, is closely associated with colorectal cancer, but the exact etiology has not yet been determined (13). UC is greater customary in developed international locations than in growing countries, however the incidence in creating nations is getting greater and higher. The gut microbiota, mucosal barrier damage, and immune response regulation are affected by nutritional imbalance and dietary factors (14).

#### 2.2.1 Environmental factor

Environmental health, lifestyle and other factors will also increase the risk of UC. Studies consistently show that smoking has an important impact on the incidence of UC (15). Compared with non-smoking, smokers have a greatly increased risk of UC, and smoking patients are usually more serious (16, 17). Studies have shown that eating vegetables and fruits can greatly reduce the risk of UC (18). On the contrary, meat consumers have a significantly increased risk of UC than vegetarian consumers, and the relationship between red meat and UC is more significant among meat consumers. In addition, drinking a large number of beverages will also increase the risk of UC (19). Alcohol can directly cause mucosal damage and increase bacterial migration, which is a risk factor for UC (20). The amount of n-3polyunsaturated fatty acids (n-3 PUFAs) in the diet is negatively correlated with UC. The more unsaturated fatty acids (n-3 PUFAs) are consumed, the lower the risk of UC. In order to maintain health, human essential fatty acids OMEGA 3 (a linoleic acid) and OMEGA 6 (oleoleic acids) are necessary, the body can not manufacture, must be obtained from the exogenous diet, but too much intake of OMEGA 3 and OMEGA 6 will increase the risk of UC (21). However, caffeine can delay the mice colitis induced by sodium glucan sulfate by down-regulating chitosinase 3-like protein 1 (22). In addition, tea is rich in tea polyphenols, which have significant effects on anti-inflammatory and antioxidant aspects, so drinking tea can reduce the risk of UC (23, 24). Gastrointestinal infections can also double the risk of UC. *Salmonella*, *Shigella*, and *Campylobacte*r motive adjustments in the intestine microbiota, which can additionally be considered in the use of antibiotics to deal with these prerequisites (25, 26).

#### 2.2.2 Individual differences and inheritance

It is estimated that 11.6 out of every 100,000 Chinese suffer from UC, according to a large number of statistical studies, especially in the southeastern coastal areas with Westernized lifestyles, North America and Europe have higher rates of UC than Asia, with 156 to 291 cases per 100,000 people (27). With the foremost onset height between the a while of 15 and 30 and between the a long time of 50 and 70, but there appears to be no specific gender preference (26, 28). In addition, IBDoften appears with hereditary familial characteristics. Modern gene detection techniques have found multiple IBD susceptible regions (such as 1p36, 21q22) and susceptible genes (such as TNFSF15, IL-23R, and IL-12B), and the multi-gene single nucleotide polymorphisms in susceptible regions suggest that they may be related to inflammation (29). The closest relationship with UC is HLA-II genes, in which DR2 and DR9 at the DR Site are considered to be susceptible genes to UC, whereas DR4 is a defensive gene and promotes the expression stage of HLA-DR antigen in the colon mucosal epithelium of UC sufferers (29, 30).

#### 2.2.3 Dysregulation of the immune response

Researchers have found that UC results in a balance to be upset between the antigen-presenting cells, the helper T cells, the regulatory T cells, and the natural killer T cells. As a result, pro-inflammatory cytokines are up-regulated and anti-inflammatory cytokines are down-regulated, which are key factors in the damage to the UC mucosa (31, 32). The production of interleukin-13 has cytotoxic effects on epithelial cells, as it induces apoptosis and alters the composition of tight junction proteins (33). Tumor necrosis factor (TNF) levels increase in patients with UC’ blood, stool samples, and mucosa (34). There is a clear link between Tumor Necrosis Factor-a (TNF-a) and UC, which confirms that TNF-a plays a significant role in its development (35, 36).

Neutrophils, as the effect cells of innate immunity, are active cells to defend against pathogen invasion. However, the unregulated mechanism of neutrophils inflammation will accelerate tissue damage and further destroy the balance of the intestinal environment. Abnormal neutrophils function can be used as early screening for chronic intestinal inflammation, and can also reflect adaptive immune function. Neutrophil infiltration in the intestinal mucosa is a key aspect of UC’s pathophysiology, with the severity of the disease being closely linked to both the degree and timing of neutrophil migration. Activated neutrophils migrate to the site of inflammation through the epithelial space and accumulate in the crypt cavity to cause crypt abscess, which is the characteristic histological and pathological change of UC (37).

CD4^+^T cells are closely linked to **UC** and can be classified into Th1 and Th2 cells in the human body. These cells play a imperative function in the immune response. The imbalance between Th1 and Th2 cells can end result in the overproduction of dangerous immune factors. This can lead to immoderate secretion of B lymphocytes, launch of antibodies, activation of the humoral immune response, complement machine activation, and infection of the intestinal mucosa. Interleukin-17 (IL-17) is secreted by Th17 cells, a subset of effector T cells. Chronic inflammation and autoimmune diseases are caused by the production of inflammatory cytokines and acute phase reactive proteins like complement C3. Unlike CD4^+^T cells, as part of the immune response, Th17 cells play an important role. The study discovered that administering a water decoction of *Rhizoma Atractylodes* had a regulatory effect on the expression levels of various Th cell related factors. Furthermore, it was observed that the levels of IL-17 in the serum of UC model rats was reduced (Table 1).

**Table 1:** Current clinical treatment for ulcerative colitis.

Treatment	Class	Example	Indication	Ref.
Modern medical treatment	Aminosalicylic acid preparation	Salazine, mesalazine, metronidazole, dexamethasone, prednisone and hydrocortisone	They can effectively improve the gut microbiota environment of patients and prevent the secretion of inflammatory cells, so as to effectively inhibit inflammation.	(48–50)
	Glucocorticoid hormones	Dexamethasone, prednisone, methylpredrone and Budesonide	They have a strong anti-inflammatory effect, has a good effect on UC, but easy to cause dependence.	(51–53)
	Immunosuppressive agents and biologics	Immunosuppressants: azathioprine, mortemycophenol, 6-mercaptopurine, cyclosporine; Biological agents: Infliximab, adalimumab; JAK inhibitor	The first choice for acute severe ulcerative colitis, overinduction and maintenance of inflammatory relief, the combination of the two is better than the single drug effect.	(54–57)
	Microbial preparation	Antibacterial drugs, probiotics, prebiotics and fecal transplants of healthy people	It can inhibit pathogenic bacteria, strengthen intestinal mucosal barrier and regulate immune response.	(58–60)
	Surgical treatment	Subtotal resection of colon, temporary ileostomy	The surgery is designed to restore the patient’s health by removing the burden on the colon, but the risk of cancer remains and requires constant testing of the rectum.	(61–63)

#### 2.2.4 Disorder of gut microbiota

Among the intestinal microbiota, the colon is the primary settlement site since it offers rich nutritional conditions to the microbiota (38). Intestinal microbiota imbalance has been shown to play a significant role in UC pathogenesis more and more studies are showing. It is bacterial growth in the human digestive tract that plays a role in the formation of mucosal barriers and maintains the stability of the intestinal environment, as well as protecting the intestinal submucosa, it triggers immune response by protecting it from gut microbiota and various toxins (39). Through metabolic products, microorganisms in the intestinal tract provide energy and nutrients to the host and regulate their immune function (40, 41).

Known as probiotics, they are live, nonpathogenic bacteria, including Lactobacillus, Bifidobacterium, and Enterococcus. Bacteria in probiotics promote gut and immune health by restoring mucosal barrier function that has been damaged, optimizing intestinal barrier function, improving gut microbiota balance, inhibiting pathogen competition, and improving immunity locally and systemically (42). It is known that probiotics secrete the most short-chain fatty acids, actic acid, butyric acid, and propionic acid. Butyrate promotes the intestinal immune system, protects intestinal epithelium integrity, inhibits tumor cell growth, and provides many other health benefits, and reduces cancer-promoting enzyme activity in the body, thereby reducing intestinal inflammation and colorectal cancer by protecting the intestinal wall (43).

*Lactobacillus* and *bifidobacterium* in *Firmicutes* and *actinomyces* of probiotics can synthesize vitamins and other substances needed by the human body, inhibits the growth of pathogenic bacteria as well. In the gut microbiota of patients with active UC, *bifidobacterium* and *lactobacillus* levels were significantly reduced, as well as the abundance of gut microbes (44). On the contrary, harmful bacteria such as *salmonella*, *clostridium difficile*, *Clostridium perfringens* and other large numbers of growth and reproduction, will cause a variety of diseases, and even produce carcinogens, if UC patients infected, will aggravate the progression of the disease and the evolution of cancer (45). Opportunistic pathogens are beneficial to human health in normal quantities, but once their proliferation is out of control or gut microbiota shifts occur, diseases such as sepsis and septic shock will occur. For example, *Escherichia coli* in *Proteobacteria*, as a normal resident bacterium in human body, can not only inhibit the growth of some intestinal microorganisms, but also reduce the harm of protein decomposition products to human body. Vitamins can also be synthesized, but if *E. coli* increases in quantity or enters the gallbladder, liver, etc., it can cause serious infection and even life-threatening (42). Pei et al. (46) found that there is a strong correlation between the ecological disturbance of the inner and outer mucous layer and the occurrence of UC, and patients with UC also have a symbiotic disturbance of mucous microflora. There is clear evidence that the gut microbiota directly influences UC occurrence, and the abnormal intestinal mucosal barrier will change the composition of pathogenic microorganisms, toxicity and invasiveness, and further aggravate inflammation. UC will also aggravate the imbalance of gut microbiota.

**Figure 1:**
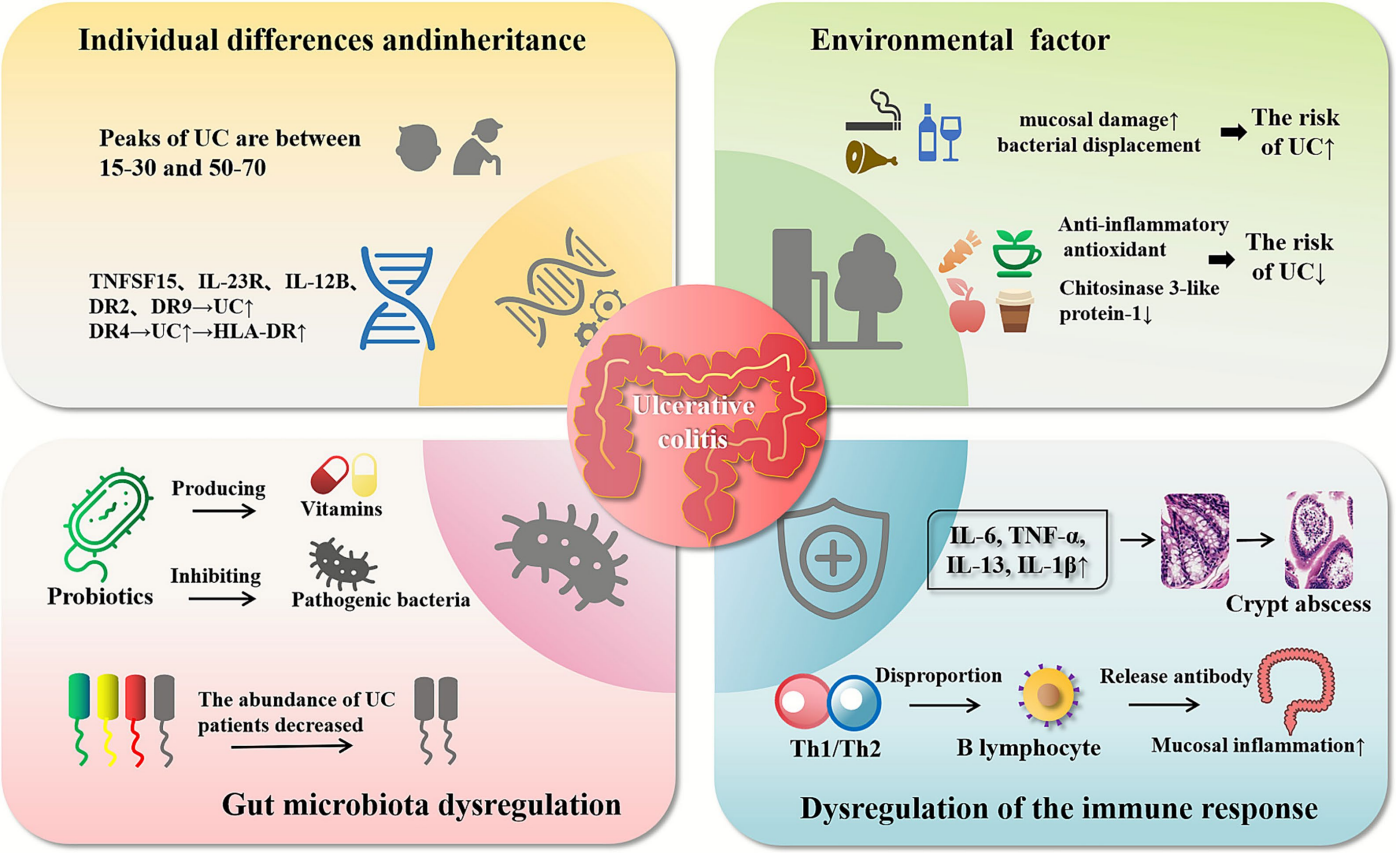
Pathogenesis of ulcerative colitis. The pathogenesis of ulcerative colitis is associated with many factors. Poor lifestyle habits and diet can lead to an increased risk of developing ulcerative colitis, and the intake of beneficial foods can reduce the incidence of UC. Ulcerative colitis occurs most often in the 15–30, 50–70 age group, and is associated with intestinal disease prone regions (e.g., 1p36, 21q22) and susceptibility genes (e.g., TNFSF15, IL-23R, and IL-12B). Ulcerative colitis causes the increase of several inflammatory cytokines (e.g., IL-6, TNF-*α*, IL-13, IL-1β). The imbalance between Th1 cells and Th2 cells may lead to the release of antibodies from B lymphocytes, causing inflammation of the intestinal mucosa. The abundance of gut microbiota, which produces vitamins and fights bacteria, decreased in UC patients.

During the long-term dynamic evolution of gut microbiota, through individual adaptation and natural selection, the dynamic balance between different species of microbiota, between microbiota and host, and between microbiota, host and environment has been formed, forming an interdependent and mutually restrictive system. When this symbiotic relationship is destroyed (such as gastrointestinal surgery, application of antibiotics, application of immunosuppressive drugs, etc.), it will cause dramatic changes in gut microbiota, leading to gastrointestinal dysfunction, and may be infected with other diseases (42, 47) (Figure 1).

### 2.3 Current clinical treatment for UC

#### 2.3.1 Aminosalicylic acid preparation

Patients suffering from mild to moderate UC are mostly treated with aminosalicylic acid preparations. It is the recommended first-line drug at the current clinical stage, and the representative drugs include sulfasalazine, mesalazine, etc. (48). Among them, sulfasalazine (SASP) is the first drug in clinical use of this kind of drug. Although more adverse reactions were found in clinical use, it is still the main drug for the treatment of UC. Some studies have explored the treatment rules of UC drugs, and found that SASP, dexamethasone, prednisone and hydrocortisone ranked the top western drugs in the treatment of UC, which further explains that SASP is a drug used more frequently (49). Patients can benefit from these drugs by improving the bacterial environment in their intestinal tract and preventing inflammation, thus effectively inhibiting inflammation. According to other relevant studies, aminosalicylic acid preparation can effectively relieve left colitis when injected locally in patients, and the clinical effect of aminosalicylic acid combined with local and oral treatment is significantly better than that of oral treatment alone (50).

#### 2.3.2 Glucocorticoid hormones

Aminosalicylic acid therapy is not effective for all patients with mild to moderate UC, that is, they fail to effectively control their clinical symptoms and prevent the progression of the disease, and when the lesions are extensive, hormone therapy can be given to these patients (51). Glucocorticoid has a strong anti-inflammatory effect, which is suitable for the treatment of moderate and severe UC, and can significantly reduce the intestinal symptoms of UC, but it is not effective in stabilizing the condition of UC. Such drugs commonly used in clinic are dexamethasone, prednisone, methylprednisolone and budesonide. The first three glucocorticoids are commonly used clinically in the past, which are mostly used to treat acute episodes of moderate and severe UC, patients with parenteral symptoms, and chronic patients who have failed other treatments (52). Most scholars recommend oral budesonide as an alternative first-line therapy for mild to moderate UC, due to its strong and effective anti-inflammatory effect and high safety, Patients with mild to moderate UC may benefit from it, as well as it has fewer adverse reactions and side effects than general glucocorticoids (53). Although hormone therapy for UC has a relatively obvious effect, but it is easy to cause hormone dependence, an appropriate dose of glucocorticoids should be determined by the severity of the disease and the individual’s tolerance for hormones.

#### 2.3.3 Immunosuppressive agents and biologics

The disorder of immune system is one of the reasons leading to the incidence of UC. In recent years, immunosuppressants are often used in clinical treatment for patients who are unable to be treated with glucocorticoid or aminosalicylic acid preparations or can not tolerate adverse drug reactions. The immunosuppressants commonly used at present include thiazolpurine, motemecol, 6-mercaptopurine, etc. Cyclosporine also has a strong immunosuppressive effect, and many scholars have pointed out that this type of immunosuppressive agent can be used as the first choice for acute severe UC. For patients with recurrence or intestinal resection, thiopurine maintenance therapy is generally used clinically (53).

Colitis is treated with TNF antibodies such as infliximab and adalimumab, reducing the need for surgical treatment of UC by inducing and maintaining inflammatory remission. Biologics have good efficacy in the treatment of UC, with high individual tolerance and few adverse reactions, which also provides another way for the treatment of UC. When it comes to clinical applications, Inflixib is the first choice. Eight-week course of infliximab in patients with moderate or severe UC, clinical symptoms were significantly relieved, mucosal healing rate was high, and during treatment, no serious adverse reactions were observed (54). A combination of immunosuppressant (azathioprine) and biologic (infliximab) is significantly more effective than either drug alone in treating UC (55).

As a new oral drug for UC treatment, JAK inhibitor acts on signal transduction in cells and reduces the production and signaling of downstream cytokines, thus down-regulating immune response and alleviating intestinal inflammatory reaction (56). Currently, JAK inhibitors under development include Tofacatinib, Felotinib, Pefitinib, etc. Tofacitinib was approved by the U. S. Food and Drug Administration (FDA) on May 30, 2018 (57). On February 20, 2023, the National Medical Products Administration of China has approved Upatinib sustained-release tablets (trade name: Refo) for the cure of suffers with moderate to severe active UC who do not respond well to or are intolerant or contraindication to one or more tumor necrosis factor (TNF) agents.

#### 2.3.4 Prebiotic preparation

For the pathogenesis and treatment of UC, a stable balance of gut microbiota and mucosa is essential. Thus, improving gut microbiota is an effective way to treat UC clinically. At present, clinical methods to improve gut microbiota mainly include antibacterial drugs, probiotics, prebiotics and fecal bacteria transplantation of healthy people (58). Probiotics are the most clinically used microecological preparations, which are a kind of bioactive microorganisms that are beneficial to the body when properly ingested. It can inhibit pathogenic bacteria, strengthen intestinal mucosal barrier, regulate immune response and so on. There has been clinical evidence that mesalazine combined with bifidobacterium triad is effective in the treatment of UC, and the results showed that the effect of the combination was higher than that of mesalazine alone (59). In order to higher recognize the relationship between intestine microbiota and UC, microecological preparations have been proposed as a new therapy. Combined with other drugs, the clinical efficacy of UC can be significantly improved, and it has been widely used in clinic (60).

#### 2.3.5 Surgical treatment

The initial stages of UC can be alleviated by medication or good lifestyle practices, but about 20 to 30% of patients with severe disease have limited response to medication, or have a sudden onset of acute UC, both of which require surgery (61). The surgical indexes of emergency patients were complicated with massive gastrointestinal bleeding, intestinal mucosa perforation, complicated with toxic paralysis, megacolon, and ineffective medical treatment. The patients undergoing elective surgery were indicated to be complicated with intestinal carcinoma. The motive of surgical treatment is to fix the patient’s fitness by means of casting off the burden on the colon, and the most important surgical strategies except getting rid of the rectal stump are subtotal colectomy and brief ileostomy (62). According to studies, people with UC who suffer from long-term disease are more likely to develop colorectal cancer (63), it is important to test the rectum continuously after surgery, even though surgery may reduce the risk of cancer for UC patients.

## 3 Human gut microbiota

### 3.1 Microbiota composition in the human gut

The gut hosts the body’s most diverse and numerous microbes, predominantly bacteria and archaea, along with fungi, viruses, and eukaryotes (64), many of which remain poorly understood.

There are more than 500 kinds, mainly in the small intestine and colon (65), which together constitute the unique mucosal system of the human body. In the stomach and small intestine, there are relatively few bacterial species in the gut microbiome, while the bacterial species in the large intestine and colon make up the majority of the gut microbiome (66).

99% of the bacteria in the gut are anaerobic, but more dense aerobic microbes are found in the cecum (67). It has been reported that *Bacteroidetes* and *Firmicutes* are the most dominant phyla in the intestinal microbiota, followed by *Proteobacteria*, *Actinobacteria*, and *Verrucomicrobia*, which collectively account for about 90% of the total human gut microorganisms (68). The most frequently recorded bacterial genera include *Bacteroides*, *Clostridium*, *Lactococcus*, *Bifidobacterium*, *Eubacterium*, *Ruminococcus*, *Faecalibacterium*, and *Streptococcus*. Of these genera, *Bacteroides* account for 30% of gut bacteria, suggesting that the physiological manifestations and functions of this genus are particularly important in the host (69). Under normal conditions, the good and bad bacteria in the biological barrier remain stable and compete for nutrients to antagonize foreign pathogens and prevent pathogenic bacteria from colonizing. An intestinal barrier is initially formed by gut microbiota. By adhering to other intestinal barriers, adhesion biological barriers prevent pathogenic bacteria and harmful substances from getting through.

### 3.2 Function of human gut microbiota

#### 3.2.1 Composition of physical and biological barriers

As food is processed and digested, there is a large area of contact between the gastrointestinal tract and the environment, and can eliminate pathogens while not affecting the survival of symbiotic microorganisms. A physical barrier is provided by epithelial cells in the gut, which serves as the gut’s main line of defense, and in addition to fighting pathogens, it limits their direct contact with epithelium by working with immune cells and stromal cells (70); Gut microbiota and intestinal mucosa are closely combined to form a biological barrier, which controls exogenous pathogenic bacteria and toxins in the intestinal lumen and inhibits their colonization and proliferation (71). The gut microbiota is also involved in the formation of the crypt structure and the differentiation of intestinal epithelial cells, thus controlling the growth of pathogenic bacteria (72). Intestinal endocrine cells and stem cells at the base of the intestinal crypt produce intestinal cells, goblet cells, Pan’s cells, and intestinal endocrine cells. It is believed that intestinal cells and Pan’s cells generate antimicrobial peptides, such as derlin, lysozyme C, and phospholipase, which play an important role in resisting pathogens (73).

However, symbiotic bacteria are not harmless and can induce severe inflammatory reactions such as colitis and septicemia if they enter the systemic immune system. Thus, reducing microbial contact with the epithelium compartments invasive gut bacteria in intestinal tissues, consequently, they are exposed to a lesser degree of systemic immunity (74). Keeping the intestinal epithelium and microbiome in limited contact is maintained by mucus secretion and processing, and the secretion of different antimicrobial factors further maintains this relationship. A lot of mucus is secreted by goblet cells, an enzyme-resistant protein that cannot be digested. A loose mucous layer covers the small intestine of mammals, which is not attached to the epithelium. This loose mucous layer sits on top of the firmly attached inner layer of mucus in the colon (75). By preventing symbiotic bacteria from penetrating the thicker inner mucus layer, this reduces contact between symbiotic bacteria and the colon’s epithelium. To prevent pathogens from infecting the host, a stable microbiome and mucous layer are essential (72).

The tight junction complex, which consists of tight junction proteins and block proteins, is another important part of the intestinal barrier mechanism, small band block proteins, and junction adhesion molecules that form a seal between adjacent intestinal cells, controlling water, ions, and nutrient permeability while blocking pathogen entry (76). Normally, tight junctions are dynamically regulated, but persistent inflammation or infection can cause adhesion molecules to be dysregulated, leading to barrier dysfunction and microbial translocation (77). As long as intestinal microbiota inhibit apoptosis and promote the synthesis of key proteins, they can improve or restore the barrier function of intestinal mucosa (78, 79). As well as secreting lactic acid and short-chain fatty acids, it also provides energy to the mucosal barrier in the intestinal tract (80).

#### 3.2.2 A component of the host’s immune system

Infection occurs primarily in the gastrointestinal tract because it serves as the primary interface between microbes and humans’ immune systems (81). As one of these functions, the microbiome plays a vital role in the host’s physiological health (82). Besides assisting in digestion and fermentation, furthermore, it competes with pathogens for nutrients and adhesion sites (81, 83). Antimicrobial peptides are even secreted by some bacteria to eliminate competition.

Adaptive and innate immune responses in the host are regulated by probiotics in the gut microbiome. Dendritic cells, monocytes, macrophages, and T and B lymphocytes are among the immune cells that these bacteria regulate, thereby enhancing their phagocytic ability to invade intestinal pathogens (84). Immunity can be affected by changes in the housing conditions of the microbial community. For example, Felix Sommer et al. found that mice without microbial community production had underdeveloped spleens, disordered lymph nodes in the B and T zones. As compared to mice raised in conventional housing, they have lower serum levels of IgG (85). The microbiome may be necessary for the immune system to develop properly.

It has been shown that Pyle’s nodes (PPs) in the small intestine and dendritic cells (DCs) in the intestinal lamina propria can remove part of the bacteria residing in the microbial community through endocytosis, which to a large extent avoids the phenomenon of bacterial overreproduction in the intestine. The symbiotic bacteria and antigen dendritic cells induce the differentiation of the original B cells to produce IgA specific to the symbiotic bacteria. These plasma cells can act as an anti-microbial immunoglobulin through efferent lymphatic vessels leaving the PPs and mesenteric lymph nodes (MLNs) and bind to the symbiotic bacteria through systemic circulation. The epithelial barrier is thus compromised, limiting their penetration (86, 87).

The vast community of commensal bacteria in the intestine necessitates a robust epithelial barrier. When this barrier is compromised, intestinal mucosal macrophages, which are derived from bone marrow stem cells, are essential for phagocytosing and clearing bacteria that penetrate into the lamina propria— a well-established immune function (88). Despite not producing pro-inflammatory cytokines, intestinal macrophages continue to phagocytose and kill bacteria (89), which greatly limits bacterial invasion into the immune system. Intestinal macrophages can also contribute to tissue repair by migrating to areas of the intestine where damage has occurred and inducing epithelial cells to proliferate and cover the damaged site (90).

#### 3.2.3 Involved in important metabolic processes of the host

Intestinal microorganisms can secrete a series of enzymes to metabolize different carbohydrates. Bacteroides and Firmicutes contain the most coding genes in the gut microbiota and are capable of utilizing polysaccharides as carbon sources (91). A bacterial protease and peptidase decompose undigested proteins into peptides, amino acids, and other metabolites. For example, *Clostridium*, *bacteroidetes* and *Lactobacillus* all have rich protease diversity (92). Intriguingly, the ratio of protein to carbohydrates determines the catabolic pathway of gut bacteria, various catabolic products can contribute to regulating the gut-brain axis or maintaining the host’s nitrogen balance (93). High-fat diets cause dysfunctional gut microbiota, decreases the abundance of gut microbiota and decreases the ratio of beneficial to harmful intestinal bacteria (94). According to Murphy et al., animals fed HFD had a decrease in Bacteroidetes and an increase in Firmicutes and Proteobacteria, the latter two being common pathogens (95). A diet high in saturated fats has also been found to increase the number of *Bacteroides* and *Bilophila*, as well as *F. prausnitzii* in the gut, while unsaturated fatty acids promote the growth of *lactobacillus*, *bifidobacteria*, and *A. muciniphila* (96), this suggests that gut microbiota is linked to diet, which means you are what you eat. A total of 17 enzymes in the liver produce bile acid, a steroid acid that helps the body remove excess cholesterol. Most of the bile acids released into the duodenum are transported to the ileum for reabsorption, while a small fraction is converted into secondary bile acids such as deoxycholate under the action of the gut microbial enzyme bile salt hydrolase (BSH) (97, 98). These secondary bile acids can be absorbed in the colon and returned to the liver via the enterohepatic circulation (99).

**Figure 2:**
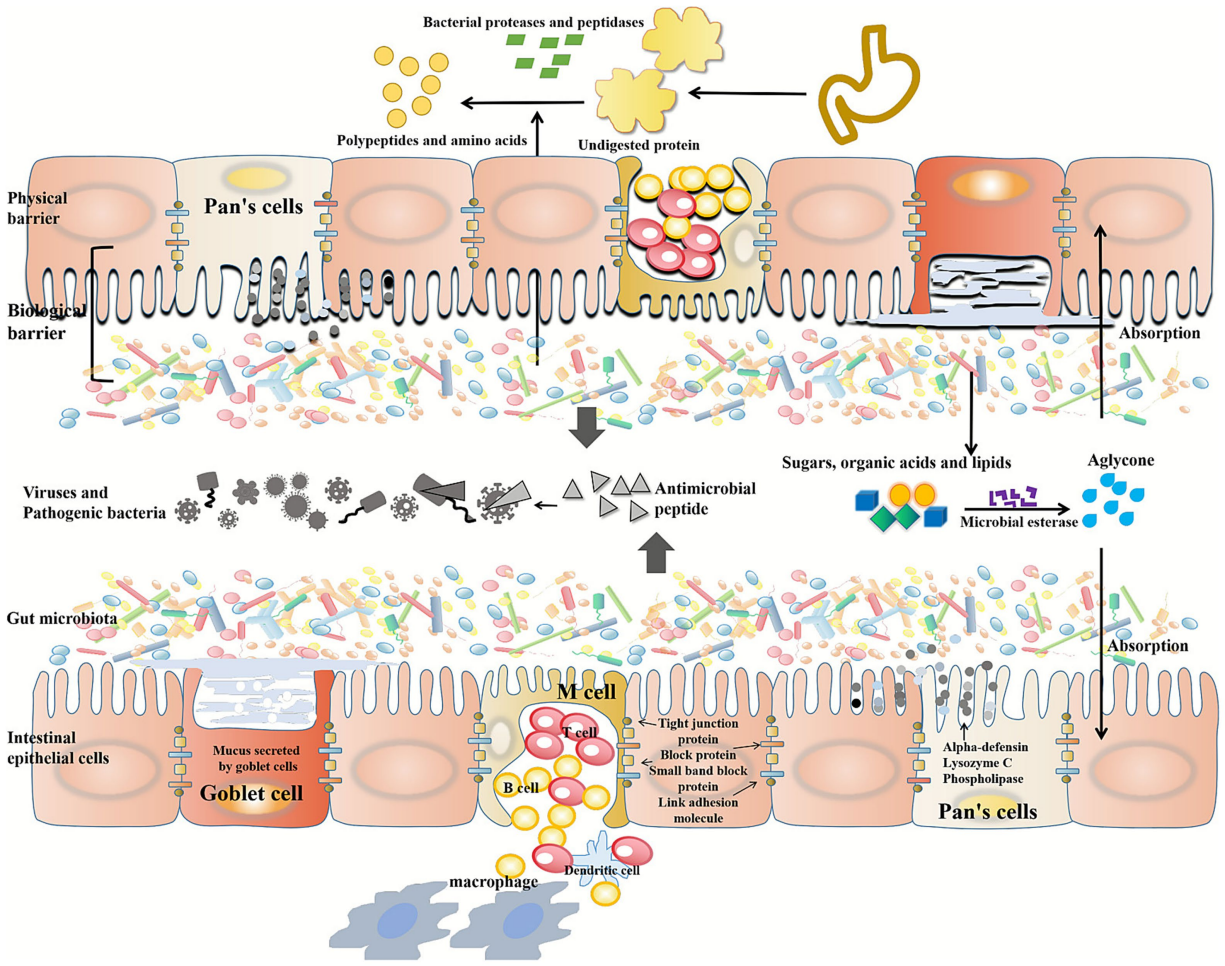
Function of human gut microbiota. Intestinal epithelial cells provide a physical barrier, a biological barrier composed of gut microbiota and intestinal mucosa. Intestinal cells are connected by tight junction proteins, block proteins, small band block proteins and junction adhesion molecules. Intestinal cells and Pan’s cells produce antimicrobial peptides, such as alpha-defensin, lysozyme C, and phospholipase, to defend against pathogen invasion. The cup cells secrete a highly glycated protein that is resistant to digestive enzymes. Gut microbes secrete a range of enzymes to metabolize different carbohydrates. In the gastrointestinal tract, undigested proteins are broken down into peptides, amino acids, and other metabolites by bacterial proteases and peptidases. Microbial esterases secreted by gut microbiota can convert sugars, organic acids and lipids into aglycans.

Furthermore, the gut microbiota can metabolize choline into trimethylamine (TMA), which the body absorbs. TMAO, a metabolite of TMAO, promotes inflammation in fat tissue and increases atherosclerosis and cardiovascular disease risk (100). Using the microbial esterase of the gut microbiota, sugars, organic acids, and lipids can be converted into aglycans. In the colon, these aglycans are absorbed and converted into hydroxyphenylacetic acid. An anti-inflammatory metabolite called urolitin A can be produced by microbial esterase from elagic acid, found in raspberries (101, 102) (Figure 2).

## 4 Association of gut microbiota with UC

### 4.1 Changes of gut microbiota structure in UC patients

As mentioned in the pathogenesis of UC, the prevention of excessive growth of microorganisms depends on the stable state of intestinal microorganisms and the balance between gut microbiota and host. In the absence of a balanced gut microbiota, the immune system, the intestinal defense function, and the body’s immunity will be impaired, and the relative pathogenic factors will increase, thus causing intestinal mucosal invasion or exacerbating diseases. There is a possibility that the imbalance of gut microbiota can damage the intestinal epithelium before UC develops, which can directly affect the intestinal permeability, so that excessive microorganisms enter the intestinal tissue and induce infection, which may lead to UC.

At present, 16S rRNA amplicon sequence analysis is commonly used in intestinal microbiome sequencing technology, which can directly show the microbial composition at the species level. However, this technology has high limitations in exploring the individual differences of patients (103). However, research designs are increasingly rationalized, including longitudinal comparisons, time-node samples, combined clinical sample analysis, etc. An analysis of 22 patients who underwent colectomy and ileobag anal anastomosis was conducted by Joseph H Vineis et al. The longitudinal microbiome of these 22 patients was studied over a two-year period and found that different bacteroides groups increased significantly when inflammation (pouchitis) was present in 9 out of 11 patients. As a model of UC, pouchitis provides prospective longitudinal studies of UC etiology before inflammation occurs. In this longitudinal study, microbial changes in UC patients are characterized before and after inflammation (104).

Fecal samples are commonly used in conventional microbiome analysis, but this approach cannot detect microbiota in specific intestinal regions. However, rising research have used endoscopic brush to learn about mucosal related microbial communities in UC and CD patients, and observed that the *α* variety of UC and CD sufferers was once notably decrease than that of non-IBD controls. The abundance of *Escherichia*, *Ruminococcus* (particularly *R. gnavus*), *Clostridium*, and *Acetobacter* was significantly higher in CD patients than in UC patients, and digestive *Streptococcus* were higher in CD patients; patients with UC had higher levels of *Enterococcus faecalis*, *Brucella*, *Bifidobacterium*, *Roseburia*, and *Citrobacter* (105). A total of 14 patients with left-sided UC or proctitis were studied by Atsushi Hirano et al., who performed mucosal biopsies at both inflammatory and non-inflammatory sites. The inflammatory site in UC patients was compared with the corresponding site in controls without IBD. The abundance of *Prevotella*, *Eubacteria*, *Neisseria*, *Leptospirosis*, *Bifidobacteria*, *sulfur-vibrio* and *butyomonas* decreased significantly (106).

Comparing germ-free mice with IL-10 or HLA-B27 knockouts with mice induced with glucans did not result in colitis compared to mice without IL-10 or HLA-B27 knockouts. Glucan-induced UC mouse models induce infection by increasing the permeability of the intestinal wall, causing intestinal bacteria to breach the barrier. There was a close relationship between gut microbiota and intestinal mucosal defense barrier and UC incidence in both studies. Fei et al. (46) found that the intestinal cavity micromicrobiota was very different from the inner mucous micromicrobiota under the condition of disease, but there was no difference under the condition of normal physiological conditions. There is an ecological imbalance between the inner and outer mucosa in patients with UC, as well as a symbiotic imbalance between the outer and inner mucosas, causing an imbalance in the mucosal microbiota. A change in gut microbiota directly affects the occurrence of selective colitis, and abnormal intestinal mucosal barrier can change the composition of pathogenic microorganisms, virulence and invasiveness, and further aggravate inflammation. UC can also aggravate the imbalance of gut microbiota.

### 4.2 Gut microbiota affects the production of intestinal metabolites in UC patients

Intestinal microorganisms produce short-chain fatty acids (SCFA) by fermenting indigestible carbohydrates, which can maintain intestinal health by promoting lipid and glucose metabolism and immune homeostasis, as well as regulating gut microbiota composition. SCFAs have been considered a class of bioactive molecules that could be used to treat intestinal inflammation in recent years. Bacteria that produce butyric acid can be used to treat IBD because of its anti-inflammatory activity. IBD patients have higher fecal microbiota volatility than healthy controls, according to long-term follow-up studies, and decreased abundance of *butyricobacteria F. peruznitzii* in the intestinal microbiota (107). Short-chain fatty acids, acetic acid, propionic acid, and valeric acid levels were significantly different between UC patients and healthy subjects, according to a meta-analysis, and in particular, in subgroup analyses, reduced SCFA levels were likely associated with disease activity, since butyric acid levels decreased significantly only during disease activity phases (108). Research on innovative drugs for IBD, as well as the clinical treatment of IBD, may benefit greatly from SCFA as a new target.

Intestinal permeability increases with impaired mucosal barrier function, allowing “attackers” to enter, thus triggering the production of local intestinal inflammation. The role of bile acids as “aggressors” in the intestinal cavity has been studied for many years to the point where no disease has been described in which bile acids are the primary initiator of injury. In spite of this, a number of studies suggest bile acids can damage the mucosal barrier of the intestinal tract and induce inflammation through relevant mechanisms. Kakiyama et al. reported that reduced gut microbial diversity and proinflammatory group expansion in patients with advanced cirrhosis were associated with reduced bile acid pools, and patients with UC experienced a similar phenomenon (109). By regulating TH17 and Treg cell balance, bile acid metabolites regulate host immune responses, according to Hang et al. (110). A study by Sinha et al. found that secondary bile acids (SBAs) reduced intestinal inflammation. The TGR5 bile acid receptor mediates this anti-inflammatory effect. An environmental dysregulation causes a SBA deficiency in patients with inflammation-prone UC, which leads to a pro-inflammatory state in the intestine, which can be treated by supplementing SBAs (111).

Intestinal microorganisms will produce aromatic amino acids under certain conditions. These active substances can promote host immune function and have anti-inflammatory and antioxidant effects on intestinal epithelium (112). Lai et al. conducted an experimental non-targeted LC/MS metabolomics analysis in patients with IBD, extracting and analyzing serum samples from both active and non-active cases, and found that most patients showed decreased serum tryptophan and tryptophan metabolites, as well as decreased ability to utilize outstanding amino acid (113). Several research have proven that tryptophan and some of its metabolites can limit the severity of colitis in mice modeled on colitis, while a bad correlation between serum tryptophan level and sickness exercise used to be determined in sufferers. Thus, tryptophan deficiency could contribute to the development of UC or aggravate the disease (114).

Inflammation is a high-energy process, and the body needs sufficient energy to maintain the normal exercise of physiological functions (115). Therefore, the energy demand in UC state increases significantly. Multiple studies on UC patients showed that citric acid, an intermediate of the tricarboxylic acid cycle (TCA), decreased in urine of UC patients (116); Serum lactate and glucose decreased, pyruvate increased. A significant increase was observed in serum acetone, acetoacetic acid, and hydroxybutyric acid in those in active disease. Serum levels of acetone, acetoacetic acid, and hydroxybutyric acid significantly declined in active, acetoacetic acid, and remission patients.

### 4.3 Application of gut microbiota in the treatment of UC

#### 4.3.1 Microecological preparation

Microecologics include probiotics, prebiotics, and synbiotics. According to the definition established by the Food and Agriculture Organization/World Health Organization (FAO/WHO), probiotics are live microorganisms that, when administered in adequate amounts, confer a health benefit on the host. The main probiotics products include *bifidobacterium*, *lactobacillus*, yeast, etc. Prebiotics are a kind of non-digestible substances, which can be decomposed and utilized by gut microbiota. As a result of their fermentation, prebiotics contribute to the growth and multiplication of beneficial intestinal bacteria, thus increasing the population of these microbes and improving intestinal function. Well-established prebiotics include lactulose, fructooligosaccharides (FOS), and galactooligosaccharides (GOS). Synbiotics, such as the commercial product Biostime, refer to a mixture of probiotics and prebiotics that work synergistically to enhance the activity and proliferation of beneficial intestinal bacteria.

Through the collection and comparison of the treatment data of 72 suffers with active UC, it was found that on the one hand, the application of probiotics in suffers with UC can regulate the gut microbiota of patients, rebuild the intestinal defense barrier structure, and improve the intestinal resistance of patients, in other words, probiotics can promote the decomposition and utilization of salazine in the intestinal cavity and increase its curative effect (117). *Bifidobacterium* triple viable powder is a common microecological preparation, but compared with simple microecological preparation, combined with immunomodulator mesalazine has better efficacy in the treatment of UC (118).

However, studies have shown that microecological preparations may lead to bacterial translocation infection, so the use of microecological preparations should be combined with the patient’s condition and psychology (119). For patients with mild disease or in the early stage and intolerant to drug side effects, combined treatment is not recommended, but single drug treatment should be selected.

#### 4.3.2 Fecal bacteria transplantation

By gavage, enema, or oral capsule, fecal microbiota transplantation is performed to transfer functional microbiota from healthy feces to patients’ intestines, so as to reconstruct gut microbiota of patients, so as to treat gastrointestinal diseases such as UC. It is possible to regulate the composition ratio of beneficial bacteria, neutral bacteria, and pathogenic bacteria in gut microbiota by transplanting feces. The efficacy of FMT is affected by individual constitution, disease severity, lesion site, FMT donor, infusion route and dose.

Eiseman et al. (120) reported the first successful application of FMT in the therapy of pseudomembranous enteritis that did not respond to conventional antibiotic therapy. Sudarshan Paramsothy et al. (121), the efficacy of FMT in UC suffers is associated with bacterial and metabolic pathways in colon mucosa and stool, and this conclusion led to the outcome of how to modify FMT therapy or more specific microbial therapy procedures. A greater diversity of donor microbiota and a greater similarity of recipient microbiota are both necessary to enhance the therapeutic effect.

Zhang et al. (122) conducted FMT treatment on 50 patients with UC, and found that FMT treatment could quickly change the intestinal microenvironment of patients with UC, improve the intestinal tract with excessive immunity, and thus alleviate the symptoms of patients with mucus, abscess, blood stool, diarrhea, abdominal pain and other symptoms. Due to the characteristics of high abundance, easy access and strong subsequent stability of the microbiota collected from FMT, FMT has the advantages of high success rate and few side effects, and it has almost become the first choice in the new treatment of UC. However, fecal bacteria transplantation still has defects and safety risks, such as non-standard operation may cause invasive infection caused by drug-resistant bacteria (71).

## 5 Summary and perspectives

UC remains a difficult-to-treat condition, with intestinal microbiota imbalance being a key pathogenic factor. All UC patients exhibit varying degrees of gut dysbiosis, and interventions targeting this state can aid recovery. However, due to the immense complexity of the gut ecosystem, the precise mechanisms and therapeutic applications of the microbiota in UC pathogenesis are not yet fully defined. This gap highlights the urgent need to bridge fundamental microbiome research with clinical practice to develop targeted therapies and improve efficacy.

The limitations of current treatments underscore the importance of focusing on systemic balance, host metabolites, and the pharmacological activity of specific gut microbes. In drug development, a compound’s effect on metabolism and the microbiota should be a critical part of pharmacodynamic evaluation. Advances in metabolomics and metagenomics are reshaping our understanding of UC, emphasizing the crucial role of metabolism and the gut microbiome—a key metabolic regulator—in maintaining host homeostasis.

Current etiological research focuses on defining specific microbial alterations, key bacterial species, and their immune mechanisms in UC. Moving forward, integrated studies—from animal models to genetic and molecular investigations—are essential. This multi-level approach is vital to elucidate pathogenesis, enable prevention, and advance microbiota-targeted treatments, ultimately aiming to modify the disease course, improve prognosis, and enhance patient quality of life.
