# Correction: The current consensus on ulcerative colitis, and the evidence and perspectives on the influence of gut microbiota on it

**DOI:** 10.3389/fmed.2026.1807286

**Published:** 2026-03-06

**Authors:** Yufeng Chen, Chang Liu

**Affiliations:** 1 The First People's Hospital of Jiashan County, Jiaxing, Zhejiang, China; 2 Reproductive Medicine Center, The First Affiliated Hospital of Wenzhou Medical University, Wenzhou, Zhejiang, China

**Keywords:** gut microbiota, inflammatory bowel disease, pathogenesis, therapeutic strategies, ulcerative colitis

The order of authors in the author list of the published paper was erroneously given as “Chang Liu and Yufeng Chen”.

The correct author list is “Yufeng Chen and Chang Liu”.

The affiliations have been re-numbered accordingly.

The original version of this article has been updated.

